# Prognostic value of nomogram model based on clinical risk factors and CT radiohistological features in hypertensive intracerebral hemorrhage

**DOI:** 10.3389/fneur.2024.1502133

**Published:** 2024-12-04

**Authors:** Gui Lu, Guodong Zhang, Jiaqi Zhang, Lixiang Wang, Baoshun Du

**Affiliations:** Department of Neurosurgery, Xinxiang Central Hospital, The Fourth Clinical Hospital of Xinxiang Medical University, Xinxiang, China

**Keywords:** clinical risk factors, CT radiologic features, nomogram model, hypertensive cerebral hemorrhage, prognostic value

## Abstract

**Objective:**

To construct a nomogram model based on clinical risk factors and CT radiohistological features to predict the prognosis of hypertensive intracerebral hemorrhage (HICH).

**Methods:**

A total of 148 patients with HICH from April 2022 to July 2024 were retrospectively selected as the research subjects. According to the modified Rankin scale at the time of discharge, they were divided into good group (Rankin scale score 0–2) and bad group (Rankin scale score 3–6). To compare the clinical data and the changes of CT radiographic characteristics in patients with different prognosis. Relevant factors affecting the prognosis were analyzed, and nomogram model was established based on the influencing factors. The fitting degree, prediction efficiency and clinical net benefit of the nomogram model were evaluated by calibration curve, ROC curve and clinical decision curve (DCA).

**Results:**

Compared with the good group, the hematoma volume in the poor group was significantly increased, the serum thromboxane 2(TXB2) and lysophosphatidic acid receptor 1(LPAR1) levels were significantly increased, and the energy balance related protein (Adropin) level was significantly decreased. The proportions of irregular shape, promiscuous sign, midline displacement, island sign and uneven density were all significantly increased (*p* < 0.05). In Logistic multivariate analysis, hematoma volume, Adropin, TXB2, LPAR1 and CT radiological features were all independent factors influencing the poor prognosis of HICH (*p* < 0.05). A nomogram prediction model was established based on the influencing factors. The calibration curve showed that the C-index was 0.820 (95% CI: 0.799–0.861), the goodness of fit test χ^2^ = 5.479, and *p* = 0.391 > 0.05, indicating a high degree of fitting. The ROC curve showed that the AUC was 0.896 (95% CI: 0.817–0.923), indicating that this model had high prediction ability. The DCA curve shows that the net benefit of the nomogram model is higher when the threshold probability is 0.1–0.9.

**Conclusion:**

The nomogram prediction model established based on hematoma volume, Adropin, TXB2, LPAR1 and other clinical risk factors as well as CT radiographic characteristics has high accuracy and prediction value in the diagnosis of poor prognosis in patients with HICH.

## 1 Introduction

Hypertensive intracerebral hemorrhage (HICH) is one of the most serious complications of hypertension, which may be due to persistent hypertension, the pathological changes such as fibrosis or hyalinization appear on the vascular wall of intracranial arterioles, and the elasticity of the vascular wall is weakened, finally leading to vascular rupture and hemorrhage (1). HICH has a rapid onset and is dangerous with a complex pathogenesis. It involves many factors, such as poor blood pressure control, arteriosclerosis, and vascular endothelial injury. Even after active treatment, there are still many patients with poor prognosis, which brings a heavy burden to the family and society (2). Therefore, in-depth study of the poor prognosis factors of hypertensive intracerebral hemorrhage is of great significance to improve the treatment effect and the prognosis of patients. According to relevant data, studies have shown that Adropin can protect the blood–brain barrier (BBB) through the Notch1 signaling pathway, and knocking down Notch1 and Hes1 *in vivo* eliminates Adropin’s protective effect. This indicates that the Notch1/Hes1 pathway plays a crucial role in Adropin’s protection of the blood–brain barrier (3). Another study has found that changes in the level of thromboxane 2 (TXB2) in cerebrospinal fluid are related to the prognosis of patients with hypertensive intracerebral hemorrhage, and the combined detection of TXB2 and S100B levels has a good predictive effect on the prognosis of patients with hypertensive intracerebral hemorrhage (4). Data shows that high levels of lysophosphatidic acid receptor 1 (LPAR1) may be associated with neurological damage and poor prognosis after cerebral hemorrhage, and therefore can serve as an important biomarker for prognostic evaluation of hypertensive intracerebral hemorrhage patients (5). CT imaging tomography is an emerging technology for comprehensive processing and analysis by extracting a large number of imaging features of tissues. In recent years, radiology has shown great potential in the evaluation of many diseases (including early hematoma enlargement of hypertensive intracerebral hemorrhage, lung cancer, and so on) (6, 7). However, due to the personal experience of the test subjects and the personal situation of the test subjects, there may be different differences. To find a prediction method with high accuracy to maximize the diagnosis and prediction efficiency of HICH prognosis is of great significance for improving the prognosis of patients. Nomogram model is a visual prediction tool, which provides a simple and intuitive method for individual prediction by integrating a variety of risk factors. In the field of medical research, nomogram models have been successfully applied to predict the prognosis of cardiovascular diseases, tumors and other diseases, and the nomogram models have high prediction accuracy, clinical practicability and operability, thus providing powerful support for clinical decision making (8, 9). In this study, we retrospectively analyzed the influencing factors of prognosis in patients with hypertensive intracerebral hemorrhage (HIH) and established an nomogram prediction model based on clinical risk factors and CT radiohistological characteristics, in order to explore its predictive value for prognosis of HIH patients.

## 2 Data and methods

### 2.1 Clinical data

A total of 148 patients with HICH were retrospectively selected as the research object, and the selection period was from April 2022 to July 2024. According to the modified Rankin scale score when patients were discharged from hospital, the prognosis of patients was evaluated, which was divided into good group (Rankin scale score 0–2 points) of 107 cases and bad group (Rankin scale score 3–6 points) of 41 cases. Inclusion criteria: (1) All patients met the diagnostic guidelines for HICH (10); (2) The onset time is within 24 h; (3) Patients with complete clinical data and normal cognitive function. Exclusion criteria: (1) patients with abnormal coagulation; (2) Disorder occurs to liver, kidney, heart and other organs; (3) Combined with other cardiovascular and cerebrovascular diseases, intracranial infection and other diseases; (4) Combined with immune system diseases.

### 2.2 Clinical data acquisition

Including gender, age, history of hypertension, hematoma location (basal ganglia, thalamus, others), hematoma volume, total cholesterol (TC), low density lipoprotein (LDL), blood pressure [systolic blood pressure (SBP), diastolic blood pressure (DBP)], energy balance related protein (Adropin), thromboxane 2(TXB2), lysophosphatidic acid receptor 1(LPAR1), etc.

Detection methods of Adropin, TXB2 and LPAR1: Enzyme-linked immunosorbent assay (ELISA) was used for detection. Preparation of samples: The cell culture medium was transferred to a sterile centrifuge tube and centrifuged at 1,000 × g for 10 min at 4 C. The supernatant was equally divided into small EP tubes and stored at −20 C (for detection within 24 h, it can be stored at 2–8 C) to avoid repeated freeze–thaw. Coating the microplate: adding a standard substance and a sample into an enzyme-labeled well pre-coated with an anti-human Adropin antibody, and after incubation, adding a biotinylated anti-Adropin antibody. Incubation and washing: incubation time is usually 90 min, followed by plate washing 2 times. Add 100 U of biotin-antibody working solution, apply the membrane, and incubate at 37°C for 60 min. Coloration reaction: The substrate TMB was added for coloration, and TMB was converted into blue under the catalysis of peroxidase. After the color reaction, the optical density was read by a microplate reader at the OD450 wavelength. Stop reaction: stop the reaction using a stop solution, usually an acidic solution. Data processing: The concentrations of Adropin, TXB2, and LPAR1 in the samples to be tested were calculated according to the standard curves.

### 2.3 CT detection

The patient took the supine position, and Innumeracy CT scanner (Philips Medical Technology Co., Ltd.) was used parameters: tube pressure: 120 kV, current: 200 mA, layer thickness: 5 mm, layer interval: 5 mm, matrix: 512 × 512. The image covered the occipital bone to the top of the head. The high-density region of interest (ROI) was traced and manually segmented on each axial slice of the CT scan using ImageJ software. To ensure that the ROI did not include high-density regions such as the surrounding bones and dura mater, PgRadiomics was used for feature extraction and Support Vector Machine (SVM) was used for feature selection, including first-order features, texture features, morphological features and wavelet features, and the intra-class correlation coefficient (ICC) of the relevant features was calculated. CT images and ROI segmentation were performed by a physician with at least 3 years of experience.

### 2.4 Statistical methods

SPSS 23.0 was used for statistical analysis. The measurement data conforming to the normal distribution were shown as (x̄ ± *s*), and the comparison between groups was examined by independent sample *t* test. Count data were expressed as [case (%)], and inter-group comparison was examined by χ^2^ test. Multivariate Logistic regression analysis was used to analyze the influencing factors. R software was used to establish an nomogram risk prediction model of multi-drug resistance for patients with diabetic foot infection, and the Bootstrap self-sampling method was used for internal verification and the calibration curve was drawn. The goodness of fit test was used to evaluate the fit of the model. The predictive value of the nomogram risk prediction model was verified by establishing a working characteristic curve (ROC) curve. The clinical net benefit of the nomographic risk prediction model was analyzed by clinical decision curve (DCA). *p* < 0.05 was considered to be statistically significant.

## 3 Results

### 3.1 Comparison of clinical risk factors between two groups

There were no significant differences between the good group and the bad group in gender, history of hypertension, proportion of hematoma location and age, TC, LDL and blood pressure (*p* > 0.05). Compared with the good group, the hematoma volume in the poor group was significantly increased, the serum TXB2 and LPAR1 levels were significantly increased, and the Adropin level was significantly decreased (*p* < 0.05). See Table 1.

**Table 1:** Comparison of clinical risk factors between the two groups [cases (%), (x̄ ± *s*)].

Group	Good group (*n* = 107)	Adverse group (*n* = 41)	*χ2/t*	*P*
Gender			1.836	0.175
Woman	47 (43.93)	13 (31.71)		
Man	60 (56.07)	28 (68.29)		
Age (years)	61.27 ± 11.30	60.86 ± 12.42	0.192	0.848
History of hypertension			0.318	0.573
Have	24 (22.43)	11 (26.83)		
Without	83 (77.57)	30 (73.17)		
Hematoma location			2.252	0.324
Basal ganglia	65 (60.75)	22 (53.66)		
Thalamencephalon	29 (27.10)	10 (24.39)		
Other	13 (12.15)	9 (21.95)		
Hematoma volume (mL)	48.79 ± 6.14	61.73 ± 7.96	10.534	<0.001
TC(mmol/L)	5.62 ± 1.14	5.74 ± 1.17	0.569	0.570
LDL(mmol/L)	2.96 ± 1.12	2.76 ± 1.07	0.984	0.327
Blood pressure (mmHg)				
SBP	129.62 ± 21.44	130.57 ± 24.84	0.231	0.818
DBP	79.20 ± 6.90	81.73 ± 7.68	1.934	0.055
Adropin(ng/L)	2.13 ± 0.30	1.72 ± 0.39	6.824	<0.001
TXB2(pg/mL)	154.27 ± 39.35	188.14 ± 42.41	4.586	<0.001
LPAR1(μmol/L)	3.27 ± 0.34	4.31 ± 0.96	9.762	<0.001

### 3.2 Analysis of CT radiographic characteristics of patients in two groups

Compared with the good group, the proportion of patients in the poor group with irregular shape, promiscuous sign, midline displacement, island sign and uneven density was significantly increased (*p* < 0.05). See Table 2.

**Table 2:** Analysis of CT radiographic characteristics of patients in the two groups [cases (%)].

Group	Good group (*n* = 107)	Adverse group (*n* = 41)	*χ2/t*	*P*
Out-of-shape			8.130	0.004
Have	40(37.38)	26(63.41)		
Without	67(62.62)	15(36.59)		
Promiscuous sign			5.087	0.024
Have	9(8.41)	9(21.95)		
Without	98(91.59)	32(78.05)		
Midline displacement			26.502	<0.001
Have	19(17.76)	25(60.98)		
Without	88(82.24)	16(39.02)		
Island sign			5.087	0.024
Have	9(8.41)	9(21.95)		
Without	98(91.59)	32(78.05)		
Non-uniform density			5.256	0.022
Have	20(18.69)	15(36.59)		
Without	87(81.31)	26(63.41)		

### 3.3 Multi-factor analysis of poor prognosis of HICH

The prognosis of HICH was taken as the dependent variable (1 = poor prognosis, 0 = good prognosis), and the statistically significant indicators were taken as the independent variables. After Logistic multivariate analysis, hematoma volume, Adropin, TXB2, LPAR1, and CT radiological characteristics were all the independent factors influencing the poor prognosis of HICH (*p* < 0.05). See Table 3.

**Table 3:** Multifactor analysis of poor prognosis of 3HICH.

Index	*B*	SE	*Wald*	*P*	*OR*	*95% CI*
Hematoma volume	0.913	0.317	8.295	0.003	2.492	1.339 ~ 4.637
Adropin	0.870	0.299	8.466	0.002	2.387	1.328 ~ 4.289
TXB2	1.124	0.356	9.969	<0.001	3.077	1.531 ~ 6.184
LPAR1	0.752	0.274	7.532	0.018	2.121	1.240 ~ 3.629
CT radiologic features	2.579	0.582	19.636	<0.001	13.184	4.212 ~ 41.264

### 3.4 Construction of nomogram prediction model

**Figure 1:**
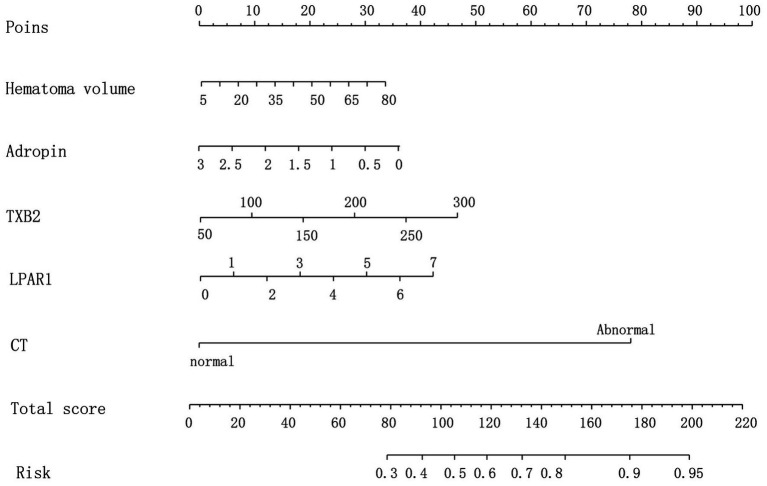
Nomogram prediction model.

Logistic multiple regression analysis was performed on the prediction of prognosis of HICH according to the R value and clinical parameters. The baseline parameter showing a higher AUC was selected as the variable for establishing the prediction model, and the corresponding specific integral value was obtained from the values of individual risk factors on the integral line at the top of the nomogram risk prediction model according to the regression coefficient (0 point corresponds to the left end point). See Figure 1.

### 3.5 Validation of nomogram risk prediction model

**Figure 2:**
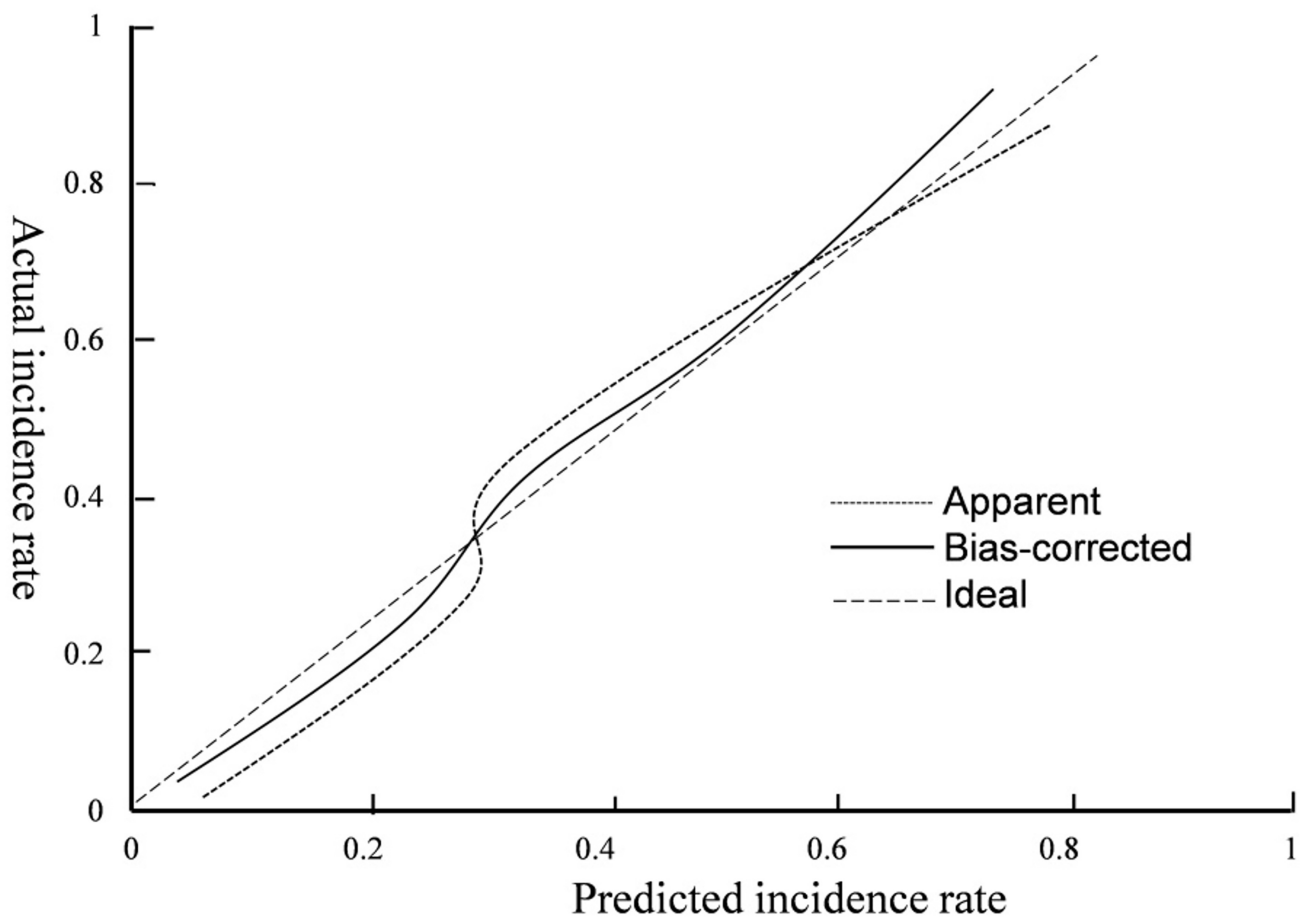
Calibration curve.

**Figure 3:**
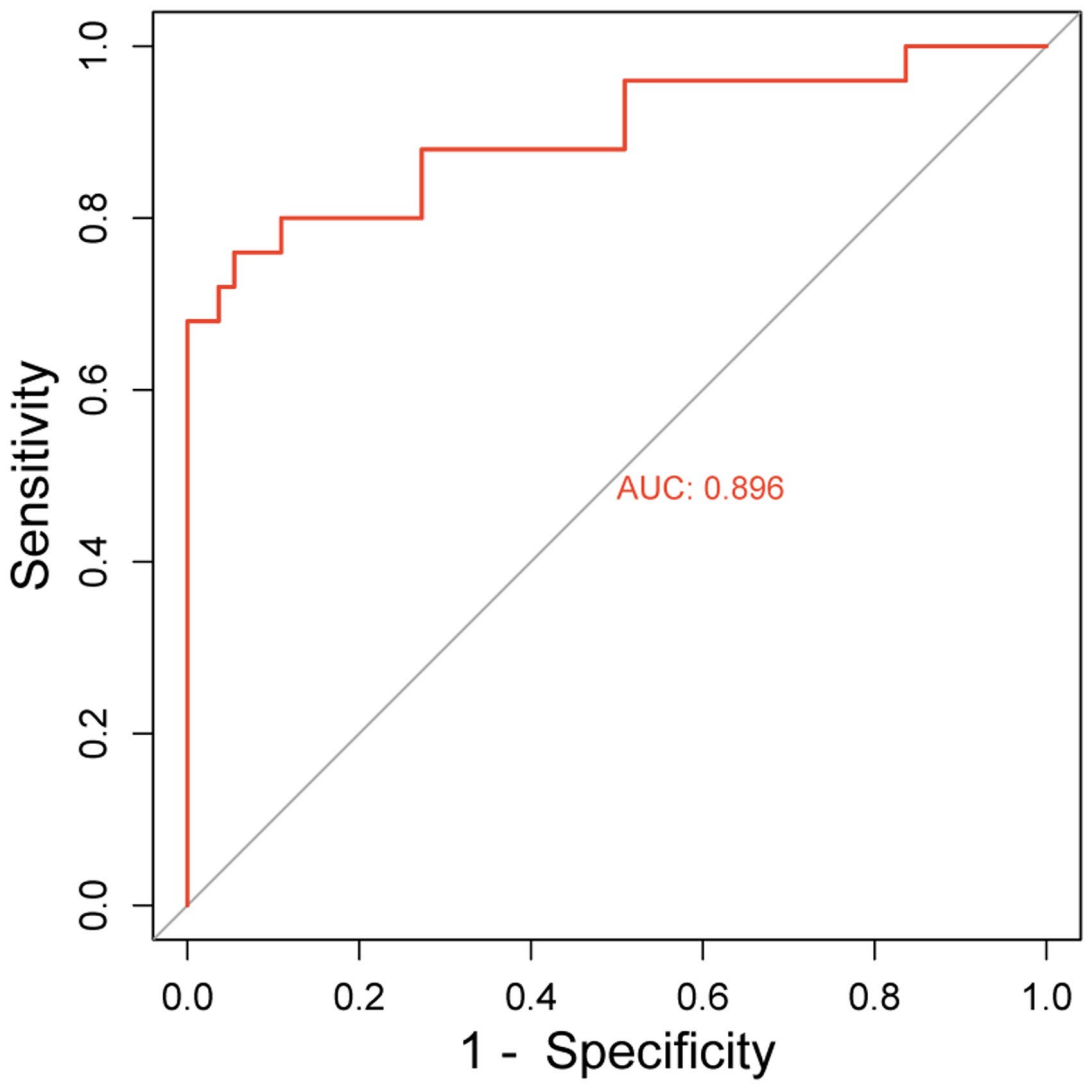
ROC curve.

**Figure 4:**
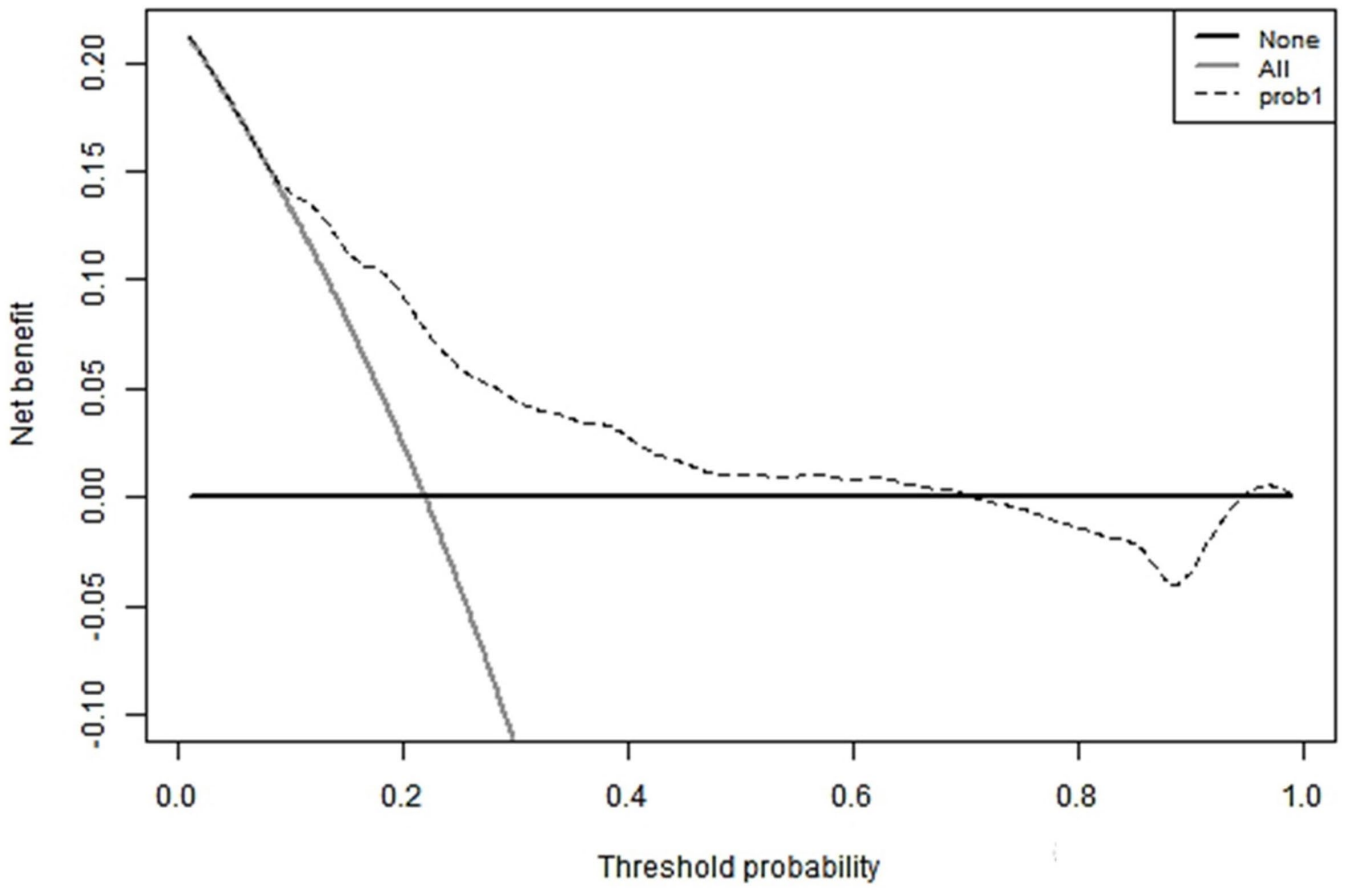
DCA curve.

The hematoma volume, Adropin, TXB2, LPAR1, and CT radiographic characteristics were used as the predictors of the nomogram model. The calibration curve showed that C-index was 0.820 (95% CI: 0.799–0.861), the goodness of fit test χ^2^ = 5.479, and *p* = 0.391 > 0.05. The calibration curve had a high degree of fit with the ideal curve (see Figure 2). The ROC curve showed that the AUC was 0.896 (95% CI: 0.817–0.923), and the nomogram prediction model had high prediction ability (see Figure 3). The DCA curve shows that the net benefit of the nomogram model is higher when the threshold probability is 0.1–0.9 (see Figure 4).

## 4 Discussion

In recent years, HICH accounts for 70 to 80% of cases of ICH, among which basal ganglia hemorrhage, as a typical manifestation of HICH, is particularly prominent (11). Although the current treatment strategies for HICH patients have become increasingly mature and efficient, early and accurate identification and intervention of those risk factors that may worsen the prognosis are still invaluable for optimizing the patient’s rehabilitation outcome. Long-term hypertension will gradually erode the cerebrovascular system, triggering the process of hyalinization and fibrosis of the microvascular wall. This pathological change not only reshapes the hemodynamic characteristics of the cerebrovascular system, but also weakens the elastic reserve capacity of the vascular wall, making the cerebrovascular system more vulnerable to blood pressure fluctuations, and finally inducing HICH (9) under the rapid changes of hypertension (12). In addition, the over-activation of the renin-angiotensin-aldosterone system (RAAS) is also considered to be an important factor behind the progression of hypertension and HICH. The imbalance of RAAS not only promotes vasoconstriction and aggravates vascular resistance, but also promotes water and sodium retention, thereby increasing the effective circulating blood volume, forming a vicious circle and aggravating the complexity of the disease and the difficulty of treatment (13, 14). An in-depth understanding of the pathophysiological mechanism of HICH, especially the dynamic evolution of primary and secondary injury, and the role of RAAS system in HICH is of profound significance for formulating more accurate and effective treatment strategies and improving the prognosis of patients.

In terms of clinical risk factors, Logistic analysis showed that hematoma volume, Adropin, TXB2 and LPAR1 were all risk factors affecting the prognosis of HICH patients. Recent studies have shown that in patients with hypertensive intracerebral hemorrhage, the hematoma volume may undergo significant dynamic changes within a period of time after hemorrhage (such as 30 min to several hours). This phenomenon of early hematoma enlargement is considered to be one of the important factors affecting the prognosis (15). Hematoma volume enlargement is common in patients with cerebral hemorrhage. The larger the hematoma volume is, the more severe the compression and destruction of the surrounding brain tissue, and thus affecting the survival and functional recovery of nerve cells (15). Studies have found that about 40% of patients with cerebral hemorrhage may have a significant increase in hematoma, and the increase in hematoma volume may have a significant impact on the survival rate, disability rate and mortality rate of patients (16). Therefore, in the treatment process of hypertensive cerebral hemorrhage, we should pay close attention to the changes of hematoma volume and take corresponding treatment measures to reduce the damage and compression of brain tissue. At the same time, other influencing factors such as bleeding site and blood pressure control should be comprehensively considered in order to formulate personalized treatment plan. Adropin is a secreted protein synthesized by the liver and brain, and has been recognized as a key regulator of cardiovascular health and metabolic balance (17). It has been found that the higher the level of Adropin, the lower the risk of cardiovascular disease, which plays an important role in regulating lipid and glucose metabolism and maintaining energy homeostasis (3). It has been reported that Adropin plays an important role in improving the neurological function of patients with cerebral hemorrhage, which may be related to the fact that Adropin promotes the appreciation of endothelial cells, microvessel formation, inhibits inflammatory reaction, and thus promotes the repair of vascular endothelial cells (18). When hypertensive cerebral hemorrhage occurs, due to the damage of vascular wall and hemodynamic changes, platelets are easily activated and release a variety of active products. TXB2 and LPAR1 are both platelet activation products, which may promote platelet aggregation and aggravate the degree of ischemia and hypoxia of brain tissue. Platelet activation products can also promote the occurrence and development of inflammatory response. After hypertensive cerebral hemorrhage, the inflammatory response is one of the important processes for the damage and repair of brain tissue. However, excessive inflammatory response may aggravate the damage of brain tissue and lead to poor prognosis (19, 20). Studies have found that in the group with poor prognosis of HICH patients, the levels of TXB2 and LPAR1 were significantly increased and correlated with the NIHSS score, which could be used as an important indicator for evaluating the prognosis of patients, with similar results to those in the present study (21, 22).

Radiomics is a field of study concerned with the extraction of quantitative features (i.e., radiomic features) from medical images that capture complex information such as tissue and lesion heterogeneity, shape, and can be used in conjunction with demographic, histological, genomic, or proteomic data to address clinical questions, and in the assessment of hypertensive cerebral hemorrhage, radiomic features may include the shape of the hematoma irregularity, mixed sign, midline displacement, island sign, uneven density, and many other indicators (23). Radiomics characterization requires only relatively simple outlining of ROIs and computer-assisted techniques to achieve the assessment of hematoma heterogeneity, which reduces the physician’s empirical requirements for the identification of imaging signs, and plays an important role in improving the accuracy of clinical diagnosis, prognosis prediction, and therapeutic efficacy, as well as facilitating clinical decision-making (24, 25). Previous studies have found that CT radiomics combined with clinical features have high value in predicting the prognosis of non-small cell lung cancer (26). In addition, CT plain imaging features have good performance in predicting hematoma volume increase in cerebral hemorrhage (27). In this study, the results found that the proportion of patients with HICH in the poor prognosis group with irregular shape, mixed sign, midline displacement, island sign, and uneven density were significantly increased, and the CT radiographic features were the risk factors affecting the patients’ poor prognosis by logistic analysis, the study will further reveal the potential value of radiographic features in evaluating the prognosis of hypertensive cerebral hemorrhage, and provide more accurate bases for clinical decision-making. The study will further reveal the potential value of radiographic features in assessing the prognosis of hypertensive cerebral hemorrhage and provide a more accurate basis for clinical decision-making.

In addition, in this study, the nomogram prediction model was established based on the relevant influencing factors. The results showed that the consistency index C-index of the calibration curve of the nomogram prediction model was 0.820, and the AUC of the ROC curve was 0.854. The model had high fitting degree and accuracy, and the net benefit value of the prediction model was higher when the range was 0.1–0.9. The above indicates that the nomogram prediction model may play a positive role in predicting the prognosis of patients with HICH. Compared with traditional clinical prediction methods, the nomogram model can more intuitively show the relationship between various risk factors and prognosis, providing a quantitative prediction tool for clinicians. In addition, the model helps to improve the accuracy of prediction and provides the basis for patients to develop individual treatment plans. In patients with HICH, targeted treatment measures can be formulated according to the relevant risk factors to adjust the treatment regimen, thereby effectively improving the prognosis of the patient. In order to reduce data collection bias, we ensure the reliability and accuracy of the data sources, rigorously check the integrity of the data, and use appropriate statistical methods to handle missing and outlier values. In order to promote research progress in this field, we have proposed several directions for future research. Firstly, we plan to conduct a large-scale prospective study to validate our findings and explore more factors related to the research topic. Secondly, we will strive to collect more comprehensive patient information, including baseline features, treatment process, and follow-up data, to further improve the accuracy and reliability of the study.

## 5 Conclusion

In summary, the nomogram prediction model based on hematoma volume, Adropin, TXB2, LPAR1 and other clinical risk factors as well as CT radiographic characteristics has high accuracy and prediction value in the diagnosis of poor prognosis in patients with HICH. This study has achieved certain results in exploring the poor prognosis of HICH patients, but there are also some limitations. Among them, the most significant is that this study used a relatively small sample size. Although we have made every effort to ensure sample diversity and representativeness, and adopted appropriate methods in statistical analysis to reduce bias, the small sample size may still limit the generalizability and robustness of the research results. Therefore, our conclusions and model predictive performance need to be validated in larger samples. To overcome this limitation and further improve the predictive accuracy of the model, we plan to adopt a multi center, multi regional approach in future studies to collect more diverse patient demographic data. This will help us build a more comprehensive and accurate model to better predict the prognosis or treatment outcomes of HICH patients. At the same time, we will also explore other factors that may affect the poor prognosis of HICH patients to further improve our research.

## Data Availability

The raw data supporting the conclusions of this article will be made available by the authors, without undue reservation.
